# Aortic Regurgitation Requiring Unplanned Surgery following Transcatheter Closure of Ventricular Septal Defect in Children: Incidence and Risk Factors

**DOI:** 10.1159/000528115

**Published:** 2022-11-22

**Authors:** Kaijun Zhang, Penghui Yang, Dan Yin, Mi Li, Xiaohua Liang, Tiewei Lv, Min Zheng, Ping Xiang

**Affiliations:** ^a^Children's Hospital of Chongqing Medical University, Chongqing, China; ^b^Department of Cardiovascular Medicine, Children's Hospital of Chongqing Medical University, Chongqing, China; ^c^National Clinical Research Center for Child Health and Disorders, Chongqing, China; ^d^Ministry of Education Key Laboratory of Child Development and Disorders, Chongqing, China; ^e^Chongqing Key Laboratory of Pediatrics, Chongqing, China; ^f^Clinical Epidemiology and Biostatistics Department, Children's Hospital of Chongqing Medical University, Chongqing, China; ^g^China International Science and Technology Cooperation Center of Child Development and Critical Disorders, Chongqing, China

**Keywords:** Ventricular septal defect, Transcatheter closure, Unplanned surgery, Aortic regurgitation, Incidence and risk factors, Children

## Abstract

**Introduction:**

Our aim was to investigate the incidence and risk factors for aortic regurgitation (AR) requiring unplanned surgery after transcatheter closure of ventricular septal defect (VSD) in children.

**Methods:**

Medical records of 876 children with VSD who underwent transcatheter closure from July 2009 to September 2018 in our hospital were retrospectively reviewed. Groups with and without new-onset or increasing AR requiring unplanned surgery were compared. Univariate and multivariate analyses were used to identify the possible risk factors. Smoothing plot and threshold effect analysis were carried out to find the relationship between possible factors and risk of new-onset or increasing AR.

**Results:**

A total of 29 children (3.3%) underwent unplanned surgery after transcatheter closure owing to new-onset or increasing AR, including 6 children with new-onset AR and 23 children with increasing AR. Multivariate regression analysis revealed that preoperative mild AR (OR: 60.39, 95% CI: 11.53–316.30, *p* < 0.001), larger ratio between diameter to body surface area (OR: 1.25, 95% CI: 1.01–1.55, *p* = 0.039), intracristal VSD (OR: 34.09, 95% CI: 4.07–285.65, *p* < 0.001), and shorter distance from the upper edge of defect to the aortic valve (or the sub-aortic rim) (OR: 0.12, 95% CI: 0.05–0.27, *p* < 0.001) were risk factors for new-onset or increasing AR requiring unplanned surgery. And, low risk of AR after muscular VSD transcatheter closure was found. An L-shaped nonlinear relationship between the sub-aortic rim and the risk of new-onset or increasing AR was observed, and the risk of new-onset or increasing AR with the sub-aortic rim up to the turning point (2 mm) (adjusted OR: 0.00, 95% CI: 0.00–0.08; *p =*0.001). With a median time of 7.3 years' follow-up, no new-onset or increasing AR has been found for children who initially did not have unplanned surgery.

**Conclusion:**

Preoperative mild AR, larger ratio between diameter to body surface area, intracristal VSD, and shorter distance of the sub-aortic rim (especially <2 mm) could increase the risk of new-onset or increasing AR requiring unplanned surgery after transcatheter closure of VSD.

## Introduction

Ventricular septal defect (VSD) is one of the most common congenital heart diseases, and surgical closure has been recommended as the standard closure strategy [1]. In recent years, transcatheter closure has been widely performed as a valuable option for selected VSD patients, particularly in developing countries, with acceptable short- and long-term complications [2, 3, 4, 5, 6, 7]. Nevertheless, unplanned surgery after transcatheter closure of VSD in children still may occur. Our previous study demonstrated that new-onset or worsening aortic regurgitation (AR) may be the primary cause for unplanned surgery [8]. Consequently, unplanned surgery owing to AR aroused our concern. Given much of the current research related to complications of transcatheter VSD closure focused on arrhythmia, the present study aimed to estimate the incidence rate and investigate the risk factors associated with AR requiring unplanned surgery after transcatheter closure through retrospective analysis. In order to decrease the likelihood of unplanned surgery owing to AR, we anticipated that the results of this study would be helpful for preoperative evaluation of transcatheter closure.

## Materials and Methods

**Fig. 1:**
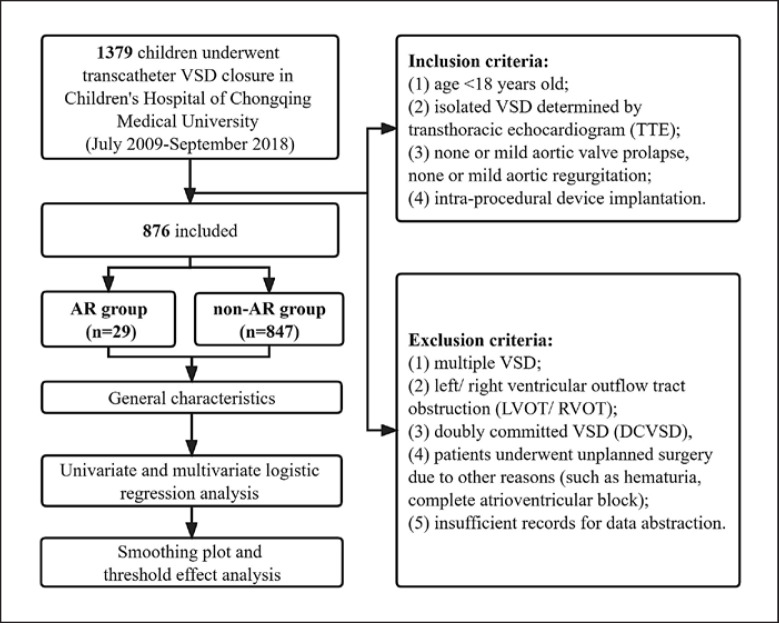
Flowchart.

Medical records of children who underwent transcatheter VSD closure in Children's Hospital of Chongqing Medical University were retrospectively reviewed from July 2009 to September 2018. Fig. 1 demonstrates a flowchart of the inclusion/exclusion of potential VSD patients along with this study's design. Inclusion criteria were (1) age <18 years old; (2) isolated VSD determined by transthoracic echocardiogram (TTE); (3) none or mild aortic valve prolapse or AR; and (4) intra-procedural device implantation. Patients with insufficient records for data abstraction, or multiple VSD, or left/right ventricular outflow tract obstruction, or doubly committed VSD, and patients underwent unplanned surgery due to other reasons (such as hematuria, complete atrioventricular block) were excluded. Enrolled children were divided into two groups according to whether unplanned surgery was performed owing to new-onset or increasing AR. Children performed unplanned surgery for AR were assigned to the AR group, while others were assigned to the non-AR group.

In our study, based on the recommendations for valvular regurgitation evaluation, AR requiring unplanned surgery was identified as a new-onset or increasing AR detected by both transthoracic color Doppler echocardiography (Philips EPIQ 7C, Philips Medical Systems) and ascending aorta angiography (Artis Q floor, Siemens Medical Solutions) during the procedure. Once a new-onset or increasing AR was detected, the procedure of transcatheter closure was interrupted, the occluder was retrieved, and an unplanned surgery would be performed [9, 10].

The legal guardian of each child has been fully informed of the potential risks of transcatheter closure and signed an informed consent form before the procedure. This study was approved by the Ethics Committee of Children's Hospital of Chongqing Medical University.

### Data Collection

We collected the patient's information from the hospital's electronic medical record system, (1) demographic information: gender, height, weight, age; (2) preoperative data: VSD diameter, VSD type, with or without aortic valve prolapses, with or without AR, with or without arrhythmia; (3) intraoperative data: VSD diameter, VSD type, pulmonary arterial systolic pressure (PASP), occluder size, occluder type, and delivery sheath size.

### Catheterization Procedure and Grading Method

Based upon standard domestic guidelines, the antegrade catheterization procedure was performed in accordance with established standard operation protocols, as previously published [11, 12, 13]. Under general anesthesia, access was obtained through the femoral vein and femoral artery. Heparin 100 UI/kg was given intravenously. The location, shape, and size of the VSD and relationship with the aortic valve were assessed by left ventriculography and TTE in all standard views. The implantation of the VSD occluder was performed as described previously. The device was deployed until the TTE result was satisfactory. Finally, left ventriculography, ascending aortic angiography, and TTE were again performed to confirm the appropriate device position, residual shunt, and valvular regurgitation.

**Fig. 2:**
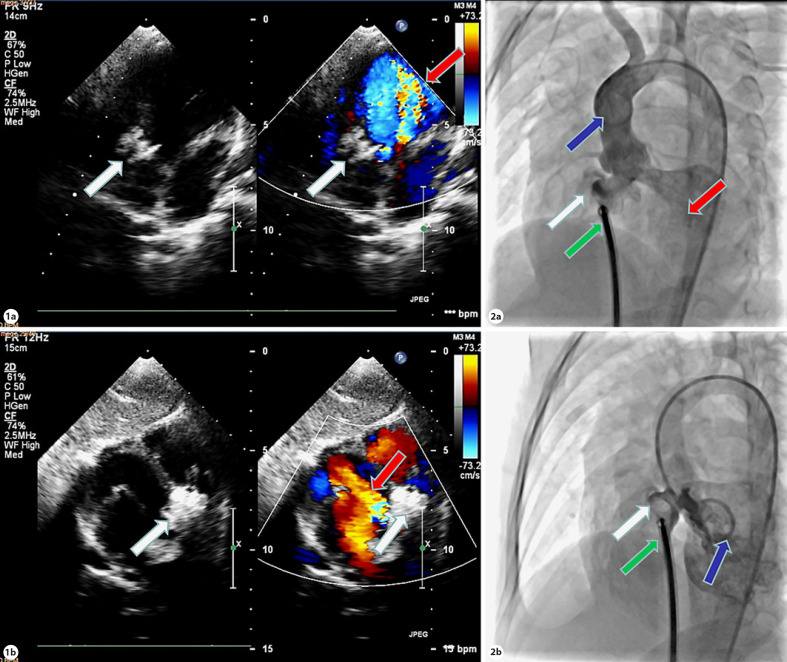
An example of a 14-kg girl with perimembranous VSD underwent conventional ascending aorta angiography in postero-anterior position, and transthoracic echocardiogram was identified as new-onset or increasing AR after occluder placing and before occluder releasing. With an 8.6-mm echo diameter, an 8.5-mm angio diameter, and 2-mm sub-aortic rim by angiography, a 12-mm symmetric occluder (Starway Medical) was selected. **1a** and **1b** Echo results. **1a** showed the massive regurgitation back to left ventricular on the five-chamber view. **1b** Regurgitation on the subcostal long-axis left ventricular view. **2a** and **2b** Angiographic results. **2a** The aortic angiography that the contrast media in blood was regurgitated back into left ventricle during the period of ventricular diastole. **2b** The left ventriculography, showing the position of the occluder. The white, red, blue, and green arrows point to the occluders, the regurgitation, the pig tail, and the sheath, respectively.

Devices with four subtypes (symmetrical, eccentric, small waist, and muscular) employed in our study were the modified double-disk VSD occluders made by LifeTech Scientific (Shenzhen, China), Shanghai Shape Memory Alloy (Shanghai, China), Starway Medical (Beijing, China) [8]. The color Doppler echocardiography and ascending aortic angiography were performed routinely to evaluate and determine whether unplanned surgery would be undertaken. Based on the ratio of the width of the AR jet to that of the left ventricular outflow tract mainly, AR was classified as mild (<0.25), moderate (0.25–0.64), and severe (≥0.65) [14]. According to the amount of the contrast regurgitation into the left ventricle during ascending aortic angiography, AR was classified as mild (1+), moderate (2+), moderate-severe (3+), and severe (4+) [9]. These two techniques essentially mirrored each other in terms of how grades were evaluated, and Fig. 2 provides an illustration.

### Follow-Up

Aspirin (3–5 mg/kg, oral, daily) was administrated to all children for 6 months. Follow-up was performed at 1, 3, 6, and 12 months at least after the procedure. Physical examination, electrocardiogram, and TTE were included as follow-up contents.

### Statistical Analysis

Categorical variables were expressed as counts and percentages and were compared by χ^2^ or Fisher's exact tests. Continuous variables were expressed as mean ± standard deviations (SD), and the Kolmogorov-Smirnov test was used for the normality test. Independent *t* tests were used for normally distributed data, while the Mann-Whitney U tests were used for data with a non-normal distribution. Univariate and multivariate logistic regression analyses were used to determine risk factors for AR requiring unplanned surgery after transcatheter VSD closure. Factors significant in the univariate analysis were subsequently included in the multivariate analysis. Smoothing plot and threshold effect analysis were performed to find the relationship between possible factors and risk of new-onset or increasing AR. *p* < 0.05 was considered as statistically significant. All tests were performed by IBM SPSS 22.0 (SPSS Inc., Chicago, IL, USA), R (http://www.R-project.org), and EmpowerStats software (www.empowerstates.com, X&Y Solutions, Inc., Boston, MA, USA).

## Results

A follow-up duration up to 12.4 years and a median time of 7.3 years were conducted. No new-onset or increasing AR, or any unplanned surgery related to cardiology has been found for those children in the non-AR group during our follow-up.

### Incidence and General Information

In our center, 1,379 children underwent transcatheter VSD closure were reviewed, and 876 children met inclusion and exclusion criteria were selected. A total of 29 out of 876 children (3.3%) underwent unplanned surgery due to new-onset or increasing AR detected by both echocardiography and angiography, including 6 children with new-onset AR and 23 children with increasing AR, all of which were done at a median time of 4 days (IQR 3–6 days) after transcatheter closure. In the AR group, 51.7% were men, the mean (SD) age was 38.82 (20.54) months, the mean (SD) weight was 14.34 (4.45) kg, and the mean (SD) body surface area (BSA) was 0.62 (0.12) m^2^. Comprehensive baseline and procedural data comparing AR group and non-AR group are listed in Table 1.

Compared to the non-AR group, patients in the AR group had a higher percentage of leaflet prolapse (69.0 vs. 12.3%), a higher percentage of preoperative mild AR (79.3 vs. 2.4%), a higher percentage of intracristal VSD (31.0 vs. 1.8%), a higher percentage of asymmetric occluder (51.7 vs. 9.6%), a higher ratio of defect diameter (angio)/BSA (7.5 ± 4.0 vs. 5.5 ± 3.0), shorter distance from the upper edge of the defect to the aortic valve (or the sub-aortic rim) (0.9 ± 1.0 vs. 3.5 ± 1.0), higher PASP (34.5 ± 11.7 vs. 30.1 ± 8.7), larger occluder size (7.4 ± 1.9 vs. 6.3 ± 1.5), and larger delivery sheath size (6.8 ± 0.8 vs. 6.3 ± 0.7) than patients did not undergo unplanned surgery (all *p* < 0.05). Moreover, compared to the patients with perimembranous VSD (pmVSD) (20/841 = 2.4%) and intracristal VSD (9/24 = 37.5%), patients with muscular VSD (0/11 = 0%) had a lower incidence of new-onset or increasing AR requiring unplanned surgery after transcatheter closure.

### Univariate and Multivariate Logistic Regression Analyses

Univariate and multivariate logistic regression analyses were performed between groups to identify risk factors for new-onset or increasing AR requiring unplanned surgery after transcatheter VSD closure (shown in Table 2). In the univariate logistic regression analysis, the following variables were found to be associated with AR requiring unplanned surgery: aortic leaflet prolapse, preoperative aortic mild regurgitation, intracristal VSD, PASP, defect diameter (angio), defect diameter (angio)/BSA, sub-aortic rim, occluder type, occlude size, and delivery sheath size. The multivariate logistic regression analysis included age, gender, weight, and factors significant in univariate analysis. In multivariate analysis, preoperative mild AR (odds ratio [OR]: 60.39, 95% confidence interval [CI]: 11.53–316.30, *p* < 0.001), higher ratio of defect diameter (angio)/BSA (OR: 1.25, 95% CI: 1.01–1.55, *p* = 0.039), intracristal VSD (OR: 34.09, 95% CI: 4.07–285.65, *p* < 0.001), and the sub-aortic rim (OR: 0.12, 95% CI 0.05–0.27, *p* < 0.001) were identified as independent and significant risk factors.

**Fig. 3:**
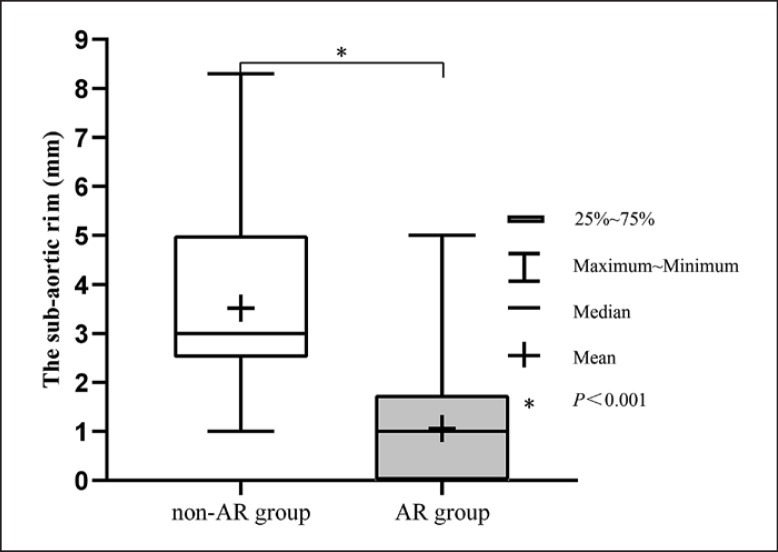
Sub-aortic rim of children without and with AR requiring unplanned surgery after transcatheter closure of VSD. *p* < 0.01.

A box plot was drawn to present the differences more intuitively of the sub-aortic rim between the two groups (shown in Fig. 3). As one of the independent risk factors, no patient with a distance less than 1 mm was fell in the non-AR group, and 1 patient with a maximum distance of 5 mm was fell in the AR group. The detailed surgical records of all those 29 patients were reviewed. There was no clear evidence of an AR association with valve impingement for 24 children, while only 5 patients with special descriptions of valve conditions were presented as follows. Patient 1, a 25-month-old boy with pmVSD, had aneurysm of membranous septum and was seen inflammatory edema at the root of the right coronary leaflet and noncoronary leaflet, which may be due to pushing up by the occluder. Patient 2, a 22-month-old boy with pmVSD, was found a 3-mm-length slit on the free edge of right coronary leaflet. Patient 3, a 36-month-old girl with pmVSD, was seen occluder clamping and inflammatory edema at the middle of the right coronary leaflet, and fibrous tissue in the right ventricle outflow. Patient 4, a 36-month-old girl with intracristal VSD, was seen occluder clamping and inflammatory edema at the middle of the right coronary leaflet. Patient 5, a 27-month-old boy with intracristal VSD, was observed obvious filamentous hyperemia at the root of the right coronary leaflet. The sub-aortic rim was 1 mm for all patients except the patient 2 (5 mm). Preoperative mild AR was only found in patients 3, 4, 5.

**Fig. 4:**
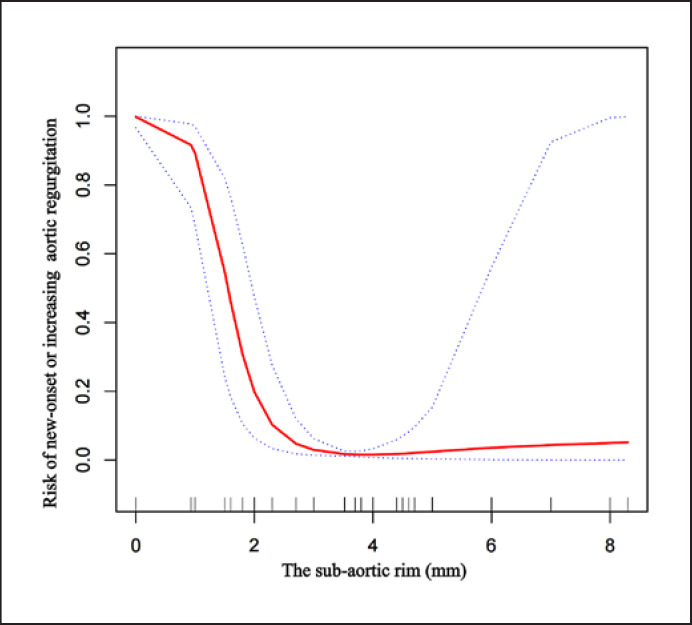
Relationship between the sub-aortic rim and the risk of new-onset or increasing AR following unplanned surgery. An L-shaped nonlinear relationship between the sub-aortic rim and risk of new-onset or increasing AR was observed after adjusting for age, sex, weight, (angio) diameter/BSA, preoperative mild AR, VSD type.

Then, an L-shaped nonlinear relationship between the sub-aortic rim and the risk of new-onset or increasing AR was observed by the smoothing plot (shown in Fig. 4). Additionally, threshold effect analysis was performed using piecewise linear regression to find its turning point (shown in Table 3). The risk of new-onset or increasing AR decreased with the sub-aortic rim up to the turning point (2 mm) (adjusted OR: 0.00, 95% CI: 0.00–0.08; *p* = 0.001). When the distance exceeded 2 mm, the distance was not associated with the risk of new-onset or increasing AR (OR: 1.11, 95% CI: 0.29–4.26; *p* = 0.871).

## Discussion

As transcatheter closure is increasingly and widely used for the treatment of VSD, complications of the transcatheter approach are gaining growing concerns [15]. Unplanned surgery, the severe situation caused by various complications associated with the transcatheter procedure, has been noted. Our recent study showed that new-onset or worsening AR might be the primary reason for unplanned surgery after transcatheter closure of VSD in children [8]. However, compared with substantial reports on arrhythmia, few studies on aortic valve structure and function damage after transcatheter VSD closure have been reported. Therefore, this study was conducted to better understand the risk factors for AR requiring unplanned surgery.

The incidence of new-onset or increasing AR requiring unplanned surgery in children following transcatheter VSD closure during the procedure was 3.3% (29/876), which may result from the leaflet distortion such as instrumental injury, leaflet propped by the occluder, leaflet clamped by the occluder, filamentous hyperemia at the root of the leaflet. Additionally, the ratio of defect diameter (angio)/BSA, the sub-aortic rim, preoperative AR, intracristal VSD were identified as risk factors for AR requiring unplanned surgery.

Transcatheter closure in children with preoperative mild AR may further aggravate the damage of aortic valve function or even AR [16]. However, preoperative mild AR may be caused by aortic valve prolapse because of the Venturi effect formed by the anatomy of VSD, so transcatheter closure of VSD could be a vital optional strategy [17]. Transcatheter closure of VSD in selected children with mild aortic valve prolapse and mild AR was performed by some interventionists, with high rates of technical success and low rates of complications [16, 18, 19, 20]. Undoubtedly, a detailed evaluation of aortic valves by echocardiogram before transcatheter closure was indispensably needed.

The higher defect diameter/BSA, shorter sub-aortic rim, and intracristal VSD interpret the relative relationship between the defect and the aortic valve from different perspectives. The difference of relative location of VSD determined the focus of preoperative evaluation and closure strategies to a great extent. Well-known, compared to perimembranous VSD and muscular VSD, intracristal VSD was closer to the aortic valve anatomically. Because of the lack of sub-aortic muscle of the ventricular septum, and contact closely between the occluder and aortic valve, the risks of secondary aortic valve injury increased after occluder placing [21]. Notably, patients with muscular VSD had a much lower incidence of new-onset or increasing AR than that of patients with pmVSD and intracristal VSD in our study, suggesting the low risk of AR after accepting muscular VSD transcatheter closure due to less chance of affecting the aortic valve, as shown in previous studies [22, 23].

The sub-aortic rim was the intuitive description of the proximity of the VSD. The shorter the distance was, the more likely the occluder interfered with the aortic valve after the double-disk occluder was deployed. The general view of Chinese medical experts is positive in performing procedures in children with a sub-aortic rim >2 mm [11]. Importantly, experts have emphasized the importance of evaluating the adjacency between the edge of the defect and the aortic valve [23, 24, 25]. In our study, a large proportion of children with AR requiring unplanned surgery was at a distance less than 2 mm. The sub-aortic rim in AR group (0.9 ± 1.0 mm) was much closer than that in non-AR group (3.5 ± 0.9 mm). Consistent with previous studies, the closure outcome of these five of the 29 children in AR group illustrated the potential impact on the aortic valve during the transcatheter procedure to a certain extent [26]. Meanwhile, our study indicated that the sub-aortic rim was associated with the occurrence of new-onset or increasing AR. More importantly, an L-shaped nonlinear relationship was found, and the risk of AR decreased with the increase of sub-aortic rim until the turning point (2 mm) had been reached. Hence, this significant importance was further confirmed that caution should be kept about transcatheter closure for children with a distance less than 2 mm for affecting the function of valves or lead AR by generating mechanical injury to the valves during the transcatheter closure process, or clamping the valves, or pushing the valves upward.

Occluder type was considered to be different in the comparison of general characteristics. Adjusted multivariate analysis did not suggest that occluder-related factors were the possible risk factors for AR requiring unplanned surgery, surprisingly, while according to our understanding and experience from other studies occluder-related factors should be associated with the occurrence of AR [15, 25, 27, 28]. This may result from the small number in AR group in our study. Moreover, we have noticed that AR may occur or increase 9–12 years after transcatheter closure, aortic valve perforation may still occur after 3 years of placement of the occluder, and AR may also progress after surgery closure [26, 29, 30]. But no aortic valve pathology was noted so far in our cohort of patients which may be secondary to the optional type of Amplatzer-sized domestic modified double-disk VSD occluder [1]. And eccentric occluder has been proven its great expectations for little effect on the function of aortic valve [3, 4, 25].

### Strengths and Limitations

This study enrolled a large sample of children with VSD (*n* = 876) in a Chinese pediatric tertiary cardiology specialty. Preoperative mild AR, intracristal VSD, higher ratio between defect diameter (angio)/BSA and shorter sub-aortic rim were found as risk factors for new-onset or increasing AR requiring unplanned surgery after accepting VSD transcatheter closure (*n* = 29) for those children, and an L-shaped nonlinear relationship between the sub-aortic rim and the risk of new-onset or increasing AR was observed. Some limitations should be considered. This was a single-center retrospective study, lacking more comprehensive perioperative variables and detailed long-term follow-up. Although cardiologists in our center strictly abided by the China Expert Consensus on VSD, the development of technology in the time span and different cardiologists may introduce confounding bias. Pulmonary to systemic blood flow ratio (Qp/Qs) was not presented. It was very unfortunate that we did not find pictures to further present the situation of the aortic valves for these five children with special descriptions of valve conditions. The advantages/disadvantages of an eccentric occluder for AR should be discussed further. And, the long-term implications of VSD occluder devices remained uncertain given that they are relatively new.

## Conclusion

In summary, with an incidence rate of 3.3%, our study concluded that new-onset or increasing AR is a relatively rare but significant complication for requiring unplanned surgery after transcatheter closure. Preoperative mild AR, intracristal VSD, higher ratio between defect diameter (angio)/BSA and shorter sub-aortic rim (especially less than 2 mm) were identified as risk factors for new-onset or increasing AR requiring unplanned surgery. And, no new-onset or increasing AR has been found in children who did not undergo unplanned surgery after a median follow-up of 7.3 years.

## Statement of Ethics

The study protocol was reviewed and approved by the Ethics Committee of Children's Hospital of Chongqing Medical University, approval number (202010). Written informed consent from parents/legal guardians for all participants aged under 18 was obtained for the publication of any potentially identifiable images or data included in this article.

## Conflict of Interest Statement

The authors declare that they have no conflicts of interest.

## Funding Sources

This study was supported by Chongqing Science and Technology Commission Social Innovation and Social Security Special Project (CSTC 2018 jscx-msybX0041).

## Author Contributions

Kaijun Zhang, Penghui Yang, Mi Li, and Ping Xiang: concept and design. Kaijung Zhang, Penghui Yang, Dan Yin, and Ping Xiang: data collection, analysis, and interpretation. Kaijung Zhang and Penghui Yang: drafting of the manuscript. Mi Li, Tiewei Lv, and Ping Xiang: administrative support, supervision, and critical revision of the manuscript. Xiaohua Liang: statistical support. Min Zheng: cardiac ultrasound support. All authors contributed to the article and approved the submitted version.

## Data Availability Statement

The original contributions presented in this study are included in the article; further inquiries can be directed to the corresponding author.

## Figures and Tables

**Table 1:** General characteristics of our study

Variable	Total (*n* = 876)	Non-AR (*n* = 847)	AR (*n* = 29)	*p* value
Gender				0.880
Male, *n* (%)	441 (50.3)	426 (50.3)	15 (51.7)	
Female, *n* (%)	435 (49.7)	421 (49.7)	14 (48.3)	
Age, months	49.78±29.82	50.15±30.02	38.82±20.54	0.086
Height, cm	102.30±16.24	102.46±16.36	97.48±11.51	0.183
Weight, kg	16.16±6.40	16.22±6.44	14.34±4.45	0.181
BSA, m^2^	0.68±0.17	0.68±0.18	0.62±0.12	0.162
Defect diameter (TTE)/BSA, mm/m^2^	9.2±3.9	9.2±3.9	9.7±3.3	0.663
Defect diameter (angio)/BSA, mm/m^2^	5.6±3.1	5.5±3.0	7.5±4.0	0.002
Defect diameter (TTE), mm	5.95±2.18	5.95±2.20	5.86±1.63	0.709
Defect diameter (angio), mm	3.62±1.82	3.59±1.80	4.45±2.02	0.018
LVEF, %	67.32±6.04	67.32±6.08	67.19±4.76	0.903
LVFS, %	36.93±3.81	36.94±3.81	36.73±3.77	0.992
Sub-aortic rim, mm	3.4±1.0	3.5±1.0	0.9±1.0	<0.001
Preoperative arrhythmia, *n* (%)	76 (8.7)	74 (8.7)	2 (6.9)	0.729
Aortic leaflet prolapse, *n* (%)				<0.001
Absent	752 (85.8)	743 (87.7)	9 (31.0)	
Prolapse	124 (14.2)	104 (12.3)	20 (69.0)	
Right coronary leaflet	59 (6.7)	50 (5.9)	9 (31.0)	
Noncoronary leaflet	33 (3.8)	31 (3.7)	2 (6.9)	
Right coronary and noncoronary leaflet	32 (3.7)	23 (2.7)	9 (31.0)	
Pulmonary hypertension, *n* (%)				
Absent	709 (80.9)	687 (81.1)	22 (75.9)	
Mild	91 (10.4)	89 (10.5)	2 (6.9)	
Moderate and above	76 (8.7)	71 (8.4)	5 (17.2)	
Preoperative mild AR, *n* (%)	43 (4.9)	20 (2.4)	23 (79.3)	<0.001
VSD type, *n* (%)				<0.001
Perimembranous/AMS	841 (96.0)/287 (32.8)	821 (96.9)/280 (33.1)	20 (69.0)/7 (24.1)	
Intracristal^a^	24 (2.7)	15 (1.8)	9 (31.0)	
Muscular	11 (1.3)	11 (1.3)	0 (0)	
PASP, mm Hg	30.2±8.8	30.1±8.7	34.5±11.7	0.006
Occluder type, *n* (%)				<0.001
Symmetric	780 (89.0)	766 (90.4)	14 (48.3)	
Asymmetric	96 (11.0)	81 (9.6)	15 (51.7)	
Muscular	28 (3.2)	28 (3.3)	0 (0)	
Eccentric	62 (7.1)	49 (5.8)	13 (44.8)	
Small waist	6 (0.7)	4 (0.5)	2 (6.9)	
Occluder size, mm	6.4±1.6	6.3±1.5	7.4±1.9	0.001
Occluder size/BSA, mm/m^2^	9.8±3.1	9.8±9.7	12.1±4.1	0.001
Sheath size, F	6.3±0.7	6.3±0.7	6.8±0.8	0.002
Sheath size/BSA, F/m^2^	9.8±2.2	9.7±2.1	11.1±2.7	0.002

TTE, transthoracic echocardiography; VSD, ventricular septal defect; BSA, body surface area; LVEF, left ventricular ejection fractions; LVFS, left ventricular fractional shortening; AMS, aneurysm of membranous septum; PASP, pulmonary arterial systolic pressure; AR, aortic regurgitation, children with new-onset or increasing aortic regurgitation; non-AR, children without new-onset or increasing aortic regurgitation; sub-aortic rim, the distance from the upper edge of the defect to the aortic valve; angio, angiography.

a A defect located at the 12:00-1:30-o'clock position in the transthoracic echocardiographic short-axis parasternal view was diagnosed as intracristal VSD.

**Table 2:** Univariate and multivariate logistic regression analyses of risk factors for new-onset or increasing AR requiring unplanned surgery after transcatheter closure

Factors	Univariate analysis		Multivariate analysis (adjusted)	
	OR (95% CI)	*p* value	OR (95% CI)	*p* value
Gender (male)	0.94 (0.45, 1.98)	0.880	−	−
Age, months	0.98 (0.97, 1.00)	0.093	−	−
Weight, kg	0.94 (0.86, 1.03)	0.182	−	−
Defect diameter (angio)/BSA, mm/m^2^	1.15 (1.05, 1.26)	0.003	1.25 (1.01, 1.55)	0.039
Sub-aortic rim, mm	0.07 (0.04, 0.14)	<0.001	0.12 (0.05, 0.27)	<0.001
Aortic right coronary leaflet prolapse	14.86 (5.65, 39.09)	<0.001	−	−
Aortic noncoronary leaflet prolapse	5.33 (1.10, 25.70)	0.037	−	−
Aortic right coronary and noncoronary leaflet prolapse	32.30 (11.73, 88.95)	<0.001	−	−
Preoperative mild AR	158.51 (58.20, 431.73)	<0.001	60.39 (11.53, 316.30)	<0.001
Intracristal VSD	24.96 (9.77, 63.76)	<0.001	34.09 (4.07, 285.65)	<0.001
PASP, mm Hg	1.04 (1.01, 1.08)	0.007	−	−
Asymmetric occluder	10.13 (4.72, 21.74)	<0.001	−	−
Occluder size/BSA, mm/m^2^	1.19 (1.09, 1.29)	<0.001	−	−

AR, aortic regurgitation; VSD, ventricular septal defect; BSA, body surface area; PASP, pulmonary arterial systolic pressure; OR, odds ratio; CI, confidence interval; angio, angiography; sub-aortic rim, the distance from the upper edge of the defect to the aortic valve. Adjusted means that gender, age, and weight were included for multivariate analysis.

**Table 3:** Threshold effect analysis of the sub-aortic rim on new-onset or increasing AR using piecewise linear regression

Models	New-onset or increasing AR			
	Crude β (95% CI)	*p* value	Adjusted β (95% CI)	*p* value
Model I				
One line slope	0.07 (0.04, 0.14)	<0.001	0.12 (0.05, 0.27)	<0.001
Model II				
Turing point (K)	3.52 (3.5^a^)		1.95 (2.0^a^)	
<K slop 1	0.05 (0.02, 0.11)	<0.001	0.00 (0.00,0.08)	0.001
>K slop 2	2.80 (1.01, 7.64)	0.048	1.11 (0.29, 4.26)	0.871
LRT test		0.002		0.002

AR, aortic regurgitation; model I, linear analysis; model II, nonlinear analysis; LRT, logarithmic likelihood ratio test. *p* value <0.05 means model II is significantly different from model I, which indicates a nonlinear relationship; crude: no adjustment; adjusted: adjusted for age, sex, weight, defect diameter (angio)/BSA, preoperative mild AR, VSD type.

a Value after taking into account the precision.
